# The Relationship between Basic Hope and Depression: Forgiveness as a Mediator

**DOI:** 10.1007/s11126-020-09759-w

**Published:** 2020-05-02

**Authors:** Kinga Kaleta, Justyna Mróz

**Affiliations:** Department of Psychology, The Jan Kochanowski University in Kielce, ul. Krakowska 11, 25-029 Kielce, Poland

**Keywords:** Basic hope, Depression, Forgiveness, Forgivingness, Mediation, Heartland, Forgiveness Scale

## Abstract

Although hope and forgiveness have been both negatively correlated with depression, actual relationships between all three variables have never been investigated. The aim of the study was to examine a theoretical model in which forgiveness mediates the relationship between basic hope and depressive symptoms. The sample was composed of 77 psychotherapy outpatients. Polish adaptations of the Basic Hope Inventory, the Beck Depression Inventory, and the Heartland Forgiveness Scale were used. Negative and positive aspects of dispositional forgiveness of self, others, and situations beyond anyone’s control were applied. Results indicated that the general level of forgiveness, as well as overcoming unforgiveness, fully mediated the relationship between basic hope and depression symptoms, while positive dimension of forgiveness partially mediated the links between the variables. The findings demonstrate that the tendency to forgive might be a mechanism via which basic hope reduces odds of depression.

## Introduction

As depression is a serious health problem, counselors and therapists have attempted to develop effective depression prevention and treatment strategies [1]. They search for new efficient interventions and therapy techniques which confer improved protection buffering against or diminishing depressive symptoms [2]. One possible psychological proceeding is an approach based on hope and forgiveness [3–5] as hope and forgiveness have both demonstrated negative associations with depression. This study tested a theoretical model in which the relationship between basic hope and depressive symptoms is mediated by dispositional forgiveness.

To explore depressive symptoms and the underlying vulnerability to depression, scholars have often focused on cognitive processing styles [6–9]. In cognitive theories, negative schemas and expectations concerning many areas of life lead to hopelessness which produces depressive symptoms [10, 11]. In turn, hopeful thinking provides positive interpretation of self, world and future [6] and might be one of important preventive variables, especially when it is conceptualized as basic hope [12]. This type of hope has been based on Erikson’s psychosocial theory and is “considered a fundamental constituent of an individual’s worldview, mostly unconscious and learned very early. It consists of the belief in two characteristics of the world: its higher order and sense and its general positivity towards a human being” [12 , p. 173]. In previous research, basic hope was negatively related to depression [12, 13]. Likewise, taking other common views of hope into account (e.g. hope for success or dispositional hope), the inverse relationship with depressive symptoms has been confirmed in various contexts [14–20]. However, psychological mechanism linking hope and depression is not clear. The role of basic hope is to stimulate and to support an individual’s constructive method of dealing with different events which pose a threat to the previous order in one’s life or in a given life aspect [12]. One of such constructive ways of coping is forgiveness, a process involving positive cognitive and emotional changes after being hurt, treated unjustly or experiencing a significant loss [21, 22]. Thus, in our model, the tendency to forgive mediates the relationship between basic hope and depression.

The proposed model might be derived from conceptualizations of forgiveness found currently in the literature. In the cognitive approach, forgiveness is about reframing the perceived harm and modifying person’s assumptions about oneself, other people and the world [21, 23]. Cognitive processes, such as placing the offense in a broader perspective, trying to understand the others’ point of view, modifying one’s previous beliefs, and forming new realistic assumptions about others and oneself, make the person see things differently [24]. An individual recovers the sense of safety and control and is able to think more positively about oneself, others, and situations. Consequently, the world becomes more comprehensible and predictable, and one’s reactions are transformed from negative to neutral or even positive. Thus, forgiveness involves not only releasing from anger, sadness, resentment, avoidance or revenge tendencies (i.e. overcoming unforgiveness), but also experience of calm, compassion, love, and benevolent thinking and motivation [25, 26].

Forgiveness conceptualized in this manner is related to both, hope and depression.

Few studies have found a positive correlation between hope and forgiveness [21, 27–29], and one revealed an inverse association between hopelessness and forgiveness [30]. However, they all used different conceptualizations of hope, such as e.g. optimism about future in different areas, hope for achieving goals or as avoidance of hope threats, and none of them have examined basic hope. Nevertheless, Mróz and Kaleta [31] revealed a positive correlation between basic hope and dispositional forgiveness, while Trzebiński and Gruszecka [32] found that basic hope was negatively related to the tendency to seek vengeance and positively related to reconciliation with the wrongdoer.

On the other hand, several studies confirmed that the higher the forgiveness, both episodic and dispositional, the lower the depression [33–35], and the higher the unforgiveness, the higher the depression [36–38]. Data obtained during intervention research also support these links [39].

## Present Study

The existing literature shows that hope and forgiveness are negatively correlated with depressive symptoms. However, we have extended the previous approach by considering all three variables in the same sample. We focused on dispositional forgiveness (called *forgivingness*) which is defined as a tendency to forgive across time, relationships and situations [40]. Also, we examined multidimensional forgiveness, namely negative and positive aspects of the ability to forgive oneself, others, and situations. Based on the existing literature, we hypothesized that basic hope and aspects of forgiveness, would be inversely correlated with level of depression.

**Fig. 1:**
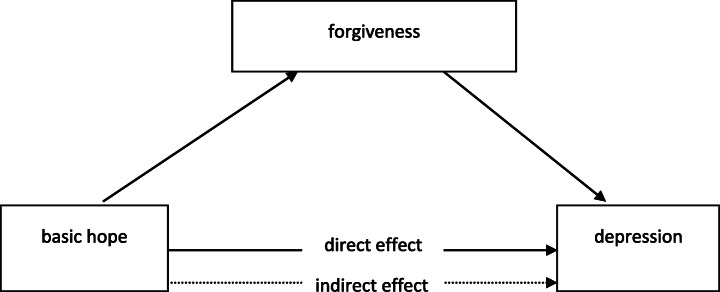
The proposed model of relationships between variables

Our second aim in the study is to examine a mediation model in which the relationships between basic hope and depression would be mediated by forgiveness. As hope is associated with improved coping [41] and forgiveness is one of coping strategies [26] enhancing mental and physical health [42], forgiveness appears to be the proper concept to describe the way in which hope might be related to reduced depression. Our expectations can be summarized in the general, conceptual model presented in Fig. 1. In the suggested model, basic hope is posited as a predictor, forgiveness – as a mediator, whereas depression as an outcome variable.

We investigated the relationship between hope, forgiveness, and depression in a sample of clinical outpatients. Although hope was negatively related to depression, for both clinical and general population [16–19], the clinical group scored lower in hope and higher in depression [19, 43]. It is consistent with the scholars’ conclusion that hope is a predictive variable, particularly in mental health patients [44]. On the other hand, clinical outpatients often experienced events that harmed them, and they may have the most to gain through forgiveness.

Summing up, the study extends the lines of previous research by addressing the need to understand mechanisms of the hope-depression relationship in a unique and understudied sample.

## Method

### Participants and Data Collection Procedure

The sample consisted of 77 psychotherapy outpatients, including 13 male and 64 female participants. At the time of data collection, all patients were involved in on-going psychotherapy. They were treated for general distress, quality of life problems, mild affective or anxiety disorders. All participants provided informed consent to participate in the study. No incentive was offered for participating in the study. Measures were completed by patients in private or during one of psychotherapy sessions, and patients were informed about their outcomes. Participants were from 21 to 65 years old, with a mean of 37.81 (SD = 9.83). The majority of respondents were married (66.2%), 1.3% widowed, 3.9% divorced, and 28.6% never married. Educational attainment ranged from vocational education (3.9%), through secondary (14.3%), and college (9.1%) to higher education (72.7%). Finally, 51.9% of the respondents lived in the cities, 5.2% lived in towns, and 42.9% in the countryside.

All participants were treated in accordance with the ethical guidelines of the American Psychological Association.

### Measures

*Basic Hope Inventory.* Hope was measured using the Basic Hope Inventory [45]. The BHI-12 is a scale measuring the strength of basic hope. It consists of 12 items, of which only 9 items are diagnostic including the following: *The world is good even if we are not doing well, The world is meaningful and all things have some sense even if we feel lost sometimes.* The inventory has a 5-point scale measuring how well a given statement expresses his or her feelings and beliefs. The higher the score (from 9 to 45 points), the higher the level of basic hope. Cronbach’s alpha reliability of the scale in the current study was .71.

*Beck Depression Inventory*. To assess depressive symptoms, we used the Beck Depression Inventory (BDI-II) [46] adapted by Polish authors [47]. It is a very popular 21-item self-report measure of the presence and severity of cognitive, affective, somatic, and motivational symptoms of depression. The BDI-II is scored on a 4-point Likert scale ranging from 0 (absence) to 3 (severe presence), which is summed to derive a total score from 0 to 63 points. Greater scores indicate higher levels of symptoms. In the current sample, mean score was 15.03 (SD = 9.24) and internal consistency was adequate (Cronbach’s alpha .88).

*Heartland Forgiveness Scale.* Disposition to forgive was measured using the Polish adaptation [48] of the Heartland Forgiveness Scale [21]. HFS is a multi-dimensional tool assessing dispositional forgiveness of self, others, and situations beyond anyone’s control. Participants rate their responses to 18 items on a 7-point scale (ranging from *absolutely false* to *absolutely true*). Sample items: *With time I am understanding of myself for mistakes I’ve made, If others mistreat me, I continue to think badly of them, I eventually make peace with bad situations in my life.* The original version consists of three subscales (forgiveness of self, forgiveness of others, and forgiveness of situations). Polish version is made of two scales (N scale and P scale) that allow to measure forgiveness in two separate domains – negative (as reduction of hostile thoughts, feelings and behaviors) and positive (as benevolent thoughts, feelings and behaviors), and six subscales with the distinction of forgiveness of self, others, and situations (N-self, N-others, N-situations, P-self, P-others, P-situations). Higher scores on each subscale reflect a higher level of forgiveness in every domain. The Total HFS score indicates how forgiving a person tends to be. Reliability and validity of the tool were satisfactory. For the present study, the values of Cronbach’s alpha (internal consistency) were as follows: .82 for overall HFS, .75 for N scale, .74 for P, .77 for N-self, .75 for N-others, .70 for N-situations, .53 for P-self, .59 for P-others, and .63 for P-situations.

## Results

Table 1 contains descriptive statistics and the Pearson correlation matrix for all variables in this study. Almost all correlations were statistically significant.

**Table 1:** Descriptive statistics and intercorrelations between analysed variables

	*M*	*SD*	1	2	3	4	5	6	7	8	9	10	11
1. Hope	31.16	4.80	–										
2. Depression	15.03	9.24	−0.39^**^	–									
3. Total forgivingness	83.42	13.53	0.50^**^	−0.56^**^	–								
4. Reduction of unforgiveness (N-scale)	39.89	8.35	0.41^**^	−0.54^**^	0.88^**^	–							
5. N-self	13.71	4.06	0.42^**^	−0.64^**^	0.59^**^	0.71^**^	–						
6. N-others	13.52	3.74	0.06	−0.07	0.52^**^	0.63^**^	0.08	–					
7. N-situations	12.66	3.86	0.39^**^	−0.42^**^	0.78^**^	0.80^**^	0.41^**^	0.31^**^	–				
8. Positive forgivingness (P-scale)	43.51	7.29	0.46^**^	−0.41^**^	0.84^**^	0.50^**^	0.29^**^	0.25^**^	0.53^**^	–			
9. P-self	15.06	2.59	0.39^**^	−0.29^*^	0.62^**^	0.36^**^	0.37^**^	0.07	0.32^**^	0.74^**^	–		
10. P-others	13.83	3.14	0.35^**^	−0.31^**^	0.76^**^	0.54^**^	0.19	0.45^**^	0.54^**^	0.79^**^	0.34^**^	–	
11. P-situations	14.62	3.37	0.35^**^	−0.38^**^	0.64^**^	0.29^**^	0.16	0.07	0.39^**^	0.86^**^	0.51^**^	0.51^**^	–

*p < 0.05; **p < 0.01

Basic hope was negatively correlated with depression and positively related to total forgiveness, reduction of unforgiveness (except for overcoming unforgiveness of others) and all positive aspect of forgiveness. Most dimensions of the disposition to forgive (except for reduced unforgiveness of others) were inversely linked to depression.

To investigate whether forgiveness mediated the linear relationship between hope and depression, we followed procedures outlined by Baron and Kenny [49] and Frazier, Tix, and Barron [50] for testing mediator effects. For mediation to be established, four conditions must be met. First, hope must be related to depression. Second, hope must be correlated with forgiveness. Third, forgiveness must be linked to depression, controlling for hope. Fourth, the association between hope and depression must be reduced. Full mediation is indicated when the predictor has no significant effect when the mediator is controlled. Partial mediation is indicated when predictor’s effect is reduced in magnitude but still significant when the mediator (forgiveness) is controlled. Requirements 1–3 for mediation are met by showing statistically significant bivariate correlations in Table 1. Hope is correlated with depression (condition one) and with forgiveness (condition two), and forgiveness is correlated with depression (condition three). Meeting of condition four requires use of multiple regression techniques. Table 2 shows hierarchical multiple regression analyses examining the mediational associations between hope, forgiveness (total score, then negative dimension and positive one), and depression. Depression was regressed onto hope (Step 1) and forgiveness (Step 2).

**Table 2:** Hierarchical regression predicting levels of depression from hope and dispositional forgiveness (total score), hope and overcoming unforgiveness (negative dimension of forgivingness), and hope and positive forgiveness (positive dimension of forgivingness)

Predictors	Predicted depression					
	*R2*	*ΔR2*	*B*	*SE*	*β*	*p*
Step 1 Hope	0.155	0.142	−0.76	0.22	−0.39	0.001
Step 2 Hope Forgiveness	0.342	0.322	−0.29 −0.35	0.22 0.08	−0.15 −0.50	0.206 0.000
Step 2 Hope N-Forgiveness	0.342	0.322	−0.40 −0.53	0.21 0.12	−0.20 −0.47	0.066 0.000
Step 2 Hope P-Forgiveness	0.223	0.199	−0.51 −0.38	0.24 0.16	−0.26 −0.29	0.035 0.020

As shown in Table 2, B representing the relationship between hope and depression was reduced to an insignificant value (from *B* = −.76 to *B* = −.29; from *β* = −.39 to *β* = − .15) when overall forgiveness (*B* = −.35, *β* = −.50, *p* < .001) was entered into the regression eq. (*R2* = .342, *ΔR2* = .322). It was also reduced to an insignificant value (from *B* = −.76 to *B* = −.40; from *β* = −.39 to *β* = − .20) when negative dimension of forgivingness (*B* = −.53, *β* = −.47, *p* < .001) was entered into the regression eq. (*R2* = .342, *ΔR2* = .322). Finally, the link between hope and depression was reduced but still significant (from *B* = −.76 to *B* = −.51; from *β* = −.39 to *β* = − .26) when positive dimension of forgiveness (*B* = −.38, *β* = −.29, *p* < .05) was entered into the regression eq. (*R2* = .223, *ΔR2* = .199).

## Discussion

Although hope has been previously proved to reduce depression, no research has explained this process to date. The aim of the study was to support the theoretical model in which dispositional forgiveness mediates the relationship between basic hope and depressive symptoms.

In line with our hypotheses, basic hope as well as aspects of forgivingness were inversely related with the level of depression. Our results are consistent with previous findings linking basic hope with depression [13], hope with dispositional forgiveness [31], and forgivingness with depression [51, 52]. Moreover, we found evidence supporting the idea that dispositional forgiveness mediates the relationship between hope and depressive symptoms. Thus, hope may operates through forgiveness to maintain a positive world-view. In case of a significant loss, conflicts or crises, they are likely to accept the situation, build up a new order, and find a constructive solution [12] which prevents emergence of depressive symptoms.

According to our results, especially the ability to overcome unforgiveness of oneself and of situations beyond anyone’s control turned out to be significant for the level of depressive symptoms. Some negative events constitute a threat to the positive self-view. Consequently, the person develops the negative self-image, i.e. that he or she does not deserve to be treated better [53], which produces depressive symptoms [54]. Alternatively, the reduction of unforgiveness, is used to overcome the negative portrayal of the self and to remove an internal barrier to relatedness caused by the transgression [53]. Thus, forgiveness breaks the vicious cycle of events related to thoughts about interpersonal rejection and depression [43, 55]. Therefore, hope-based forgiveness might be seen as a protective factor in self-deprecatory thinking [41], which is the cognitive cause of depression [10, 11]. Our findings are consistent with those from the study by Chang, Kahle, Yu and Hirsch [56] who also found that forgiveness of self, but not forgiveness of others, accounted for (that is, eliminated) the positive link between domestic abuse and suicide behavior. As both studies were conducted among outpatient, this may be especially important for mental health professionals to assist in facilitating forgiveness of self in their clients. Many clinical outpatients experienced events that harmed them and violated their personal order. Therapist may help them by focusing on understanding their personal choices, relieving feelings like self-blame, and promoting the motivation to change. Thus, increased forgiveness of self should mitigate maladaptive cognitions that have been correlated with depression. Summing up, hope-based forgiveness provides a cognitive framework for understanding hope experience and development in terms of their capacities to reduce negative thoughts about oneself and the world, and evoking positive thinking about them.

It is important to note some limitations of the present study. Firstly, it was a cross-sectional study, therefore the causal pathway was not possible to be determined. Future research should consider searching for alternative paths and a mechanism to account for the hope and forgiveness relationship. A possible alternative model might stipulate that forgiveness reduces levels of depression by increasing hope. Another possible hypothesis might examine the statement that depression reduces forgiveness [57] which in turn diminishes hope. Investigating these possibilities may provide a conceptual model of how the three variables are associated. A longitudinal assessment to further describe the direction of the causal relationship is also suggested. Secondly, there are many potential factors in the hope-depression relationship that were not included in the analyses, which may affect the level of depressive symptoms, such as sociodemographic variables, clinical diagnosis, history of treatment, including or not including pharmacotherapy. A tendency to forgive is also related to age, gender or other personality traits [58, 59]. It would be beneficial to examine these variables in more complex models to specify a chain of distal and proximal contributory causes of depression levels. Thirdly, a clinical sample was used in this study, and as a rule it is special and small. To improve generalizability, studies must gradually include other populations, such as individuals who experienced particular difficulties, such as a recent breakup, assault, transport accident or lawsuit, as well as non-clinical samples.
